# Current Concept and Update of the Macrophage Plasticity Concept: Intracellular Mechanisms of Reprogramming and M3 Macrophage “Switch” Phenotype

**DOI:** 10.1155/2015/341308

**Published:** 2015-08-23

**Authors:** Igor Malyshev, Yuri Malyshev

**Affiliations:** ^1^Moscow State University of Medicine and Dentistry, Delegatskaya Street 20/1, Moscow 127473, Russia; ^2^Institute of General Pathology and Pathophysiology, Baltijskaya Street 8, Moscow 125315, Russia; ^3^University of North Texas Health Science Center, 3500 Camp Bowie Boulevard, Fort Worth, TX 76107, USA; ^4^Moscow State University, GSP-1, Leninskie Gory, Moscow 119991, Russia

## Abstract

Macrophages play a key role in immunity. In this review, we consider the traditional notion of macrophage plasticity, data that do not fit into existing concepts, and a hypothesis for existence of a new switch macrophage phenotype. Depending on the microenvironment, macrophages can reprogram their phenotype toward the proinflammatory M1 phenotype or toward the anti-inflammatory M2 phenotype. Macrophage reprogramming involves well-coordinated changes in activities of signalling and posttranslational mechanisms. Macrophage reprogramming is provided by JNK-, PI3K/Akt-, Notch-, JAK/STAT-, TGF-*β*-, TLR/NF-*κ*B-, and hypoxia-dependent pathways. Posttranscriptional regulation is based on micro-mRNA. We have hypothesized that, in addition to the M1 and M2 phenotypes, an M3 switch phenotype exists. This switch phenotype responds to proinflammatory stimuli with reprogramming towards the anti-inflammatory M2 phenotype or, contrarily, it responds to anti-inflammatory stimuli with reprogramming towards the proinflammatory M1 phenotype. We have found signs of such a switch phenotype in lung diseases. Understanding the mechanisms of macrophage reprogramming will assist in the selection of new therapeutic targets for correction of impaired immunity.

## 1. Introduction

The immune system recognizes and eliminates pathogenic microbial products and tumor cells, thus preventing the progression of many diseases. Macrophages play a crucial role in this system. Among all the immune cells, macrophages have the greatest plasticity [1–3]. In various tissues, macrophages acquire their long-term, respective phenotype [4] such as Kupffer cells in the liver, alveolar macrophages in the lungs, microglia in the brain, osteoclasts in bone tissue, Langerhans cells in the skin, adipose tissue macrophages in adipose tissue, and peritoneal macrophages in the peritoneal cavity.

Macrophages are the sensor cells of the immune system. They identify pathogenic cells with pattern recognition receptors (PRR), such as Toll-like receptors (TLR) [5], and water-soluble pattern recognition molecules (PRM), such as ficolins and collectins [6]. PRR identify different microbe-specific molecules, for example, bacterial carbohydrates such as lipopolysaccharide (LPS) and zymosan from the cell wall of yeast. In response to the binding, PRR activate intracellular signalling pathways of immune reactions. PRM represent evolutionarily ancient antibodies. They play an important role in the neutralization of such microbes as* Aspergillus fumigates* and* Pseudomonas aeruginosa*.

Macrophages, together with dendrite cells, function as antigen-presenting cells (APC), whereas, together with natural killer cells (NK) and T- and B-lymphocytes, they eliminate microbes and tumor cells. In addition, macrophages participate in tissue repair and remodelling, angiogenesis, and restoration of pathogen-disturbed homeostasis [4, 7]. The efficacy of immune defence depends on the extent of macrophage plasticity, that is, on how quickly and adequately the cells can change their functional phenotype in response to the microenvironment and pathogenic microbes.

Disturbances in macrophage plasticity might compromise immune responses and the development of a number of diseases, such as cancer, bronchial asthma, and atherosclerosis. Therefore, a more complete understanding of the mechanisms responsible for disorders of macrophage plasticity that affect macrophage functional phenotypes is critical for advances in immunology, pathophysiology, and medicine.

In this review, key phenomena and mechanisms of macrophage plasticity will be addressed. In addition, we will consider published experimental data that do not conform to the current concept of macrophage plasticity. These data lead us to hypothesize a new macrophage phenotype, which we name the switch phenotype.

## 2. Current Concept of Macrophage Plasticity

### 2.1. Macrophage M1 And M2 Phenotypes: The Concept of a Continuum and Criticism of Some Aspects

In response to viral or bacterial infection, macrophages produce proinflammatory cytokines such as IL-12 and TNF-*α* and chemokines [8–10], which attract neutrophils, NK, and Th- and T-lymphocytes [11]. By affecting NK and macrophages, IL-12 and TNF-*α* stimulate IFN-*γ* secretion, which, in turn, results in a further increase in IL-12 and TNF-*α* production by macrophages, thus enhancing their bactericidal, antiviral, and antitumoral activities. When parasites such as fungi or helminthes are recognized, macrophages produce anti-inflammatory cytokines, including IL-4, IL-10, IL-13, and TGF-*β* [8, 10]. These chemokines attract Th-lymphocytes, eosinophils, and basophils that produce IL-4 and IL-13 [12], which, in turn, further stimulate IL-10 secretion by macrophages [12]. IL-10 suppresses the production of proinflammatory cytokines [13], reactive oxygen species (ROS), and nitric oxide (NO). This reduces the bactericidal properties of macrophages and enhances their antiparasitic properties.

The proinflammatory phenotype formed under the influence of LPS and/or IFN-*γ* was termed M1, and the anti-inflammatory phenotype formed under the influence of IL-4, IL-13, and IL-10 was termed M2 [14, 15]. The M1 phenotype is characterized by TLR-4, the MARCO receptor, CD25, and CD80. The markers of the M2 phenotype include the mannose receptor, SR-A, CD163, CD209, and FIZZ1 [15].

The current concept of macrophage plasticity postulates that proinflammatory factors, such as LPS and IFN-*γ*, program the M1 phenotype to enhance the production of proinflammatory cytokines. These cytokines shift the macrophage phenotype even further towards M1. As a result, a proinflammatory positive feedback mechanism is formed, enabling fast programming of the antimicrobial and antitumoral M1 phenotype. Similarly, anti-inflammatory cytokines, such as IL-10, IL-13, or TGF-*β*, program the M2 phenotype, which then intensively produces more anti-inflammatory cytokines. These cytokines shift the macrophage phenotype even further towards the M2 type, thus again forming an anti-inflammatory positive feedback mechanism, enabling the fast programming of the antiparasitic M2 phenotype of macrophages [8, 10].

When the microenvironment or active pathogen changes, tissue macrophages can change their functional phenotype [8, 9, 16]. The process of changing the macrophage phenotype is called* reprogramming*. This process is also frequently referred to as* polarization* or* alternate phenotype*.* Polarization* and* alternate phenotype* assume a choice from two states. It was clear from the very beginning that these terms do not reflect the real nature of reprogramming. Therefore, additional phenotypes, М2a, M2b, and M2c, were recognized, and the concept of a continuum soon appeared [1, 17, 18]. This concept implies that macrophage activation varies along a continuous proinflammatory spectrum: early stage M1 macrophages and later stage anti-inflammatory M2 macrophages. It was postulated that each phenotype is formed in response to action of certain inductors: M1 in response to LPS and/or IFN-*γ*; М2а in response to IL4/IL13; M2b in response to immune complexes; M2c in response to IL-10 or TGF-*β* [18–21]. It is important to note that, under* in vivo* conditions, there are usually several different reprogramming factors that affect macrophages simultaneously. For example, those can be LPS, IL-4, TGF-*β*, and immune complexes. Which phenotype is formed in such a case, and how should it be referred to? Should it be referred to as М1/2аbс? Obviously, identifying the macrophage phenotype according to its reprogramming factor is not practical because (1) the same inductor, for instance, LPS or hypoxia, is able to program both the M1 and M2 phenotype depending on its concentration or extent [22–24]; (2) there are many reprogramming factors, which can act in different compositions; (3) as more new reprogramming factors are discovered, no more letters in the English alphabet may be available for identifying the new phenotype.

There are at least three categories of reprogramming factors. Among them are the following: (1) immune factors, such as immune complexes and cytokines [25]; (2) pathogen-associated molecular patterns (PAMP), such as LPS, microbial nucleic acids, proteins, or carbohydrates that can be recognized by PRR of macrophages; (3) physical and chemical factors, such as hypoxia [26], fever, and pH [27]. It has recently been proposed that factors which shift macrophage phenotype towards M1 be referred to as reprogramming factor- (RF-) M1 and those which shift the phenotype towards M2 as RF-M2 [28]. For instance, RF-M1 is used for IFN-*γ* and low concentrations of serum [29], and RF-M2 is used for IL-4 and high concentrations of serum [14].

To date, no pure macrophage phenotype having only M1 or M2 markers has been described. Therefore, it would be correct to consider the M1 phenotype as having more M1 than M2 markers and vice versa. Thus,* reprogramming* is more appropriate for describing the formation of any cell phenotype, whereas the terms* polarization* and* alternate phenotype* should be used to describe only the pure phenotypes, which, strictly speaking, do not exist. Therefore, we will use the term* reprogramming* rather than* polarization* or* alternate phenotype*.

The difference between macrophage phenotypes is most obvious during macrophage activation in response to the same stimulus, as demonstrated for the first time by Morrison [22, 23]. In particular, it was shown that the preliminarily reprogrammed (preprogrammed) M1 and M2 macrophages produced different amounts of cytokines and NO in response to the same dose of LPS.

Reprogrammed macrophages and APC activate the cellular or the humoral type of the immune response. When the cellular type of the immune response occurs, APC and M1 macrophages stimulate Th0 cells to differentiate into Th1 cells and T cells into CTL (cytotoxic T-lymphocytes). M1 macrophages, Th1, and CTL kill bacteria, viruses, and tumor cells [30, 31]. When the humoral type of the immune response occurs, APC and M2 macrophages stimulate Th0 cells to differentiate into Th2 cells [32]. M2 macrophages, together with Th2 cells, remove parasites and toxins by releasing IL-4, which promotes the activation of B-cells and the production of antigen-specific antibodies [8, 33]. In addition, M2 macrophages participate in tissue repair, angiogenesis, and phagocytosis of apoptotic cells [34–36].

Thus, the reprogramming of macrophages causes changes not only in the functioning of the macrophages themselves but also in the functioning of other immune cells, thus providing the required plasticity of the immune response required for eliminating pathogenic factors.

### 2.2. The Macrophage Reprogramming Phenomena

The macrophage reprogramming process is characterized by four phenomena. 

*(1) The Phenomenon of an Amplified Macrophage Response to Both the Reprogramming Factor (Direct Amplification) and Another Factor (Cross Amplification).* For instance, the reprogramming due to IFN-*γ* enhances the following macrophage response to IFN-*γ* itself (direct amplification) and to LPS (crisscross amplification).

*(2) The Phenomenon of Reciprocal Suppression of the Alternate Phenotype.* M1 phenotype reprogramming enhances production of proinflammatory cytokines while suppressing production of anti-inflammatory cytokines and formation of the M2 phenotype. M2 phenotype reprogramming enhances production of anti-inflammatory cytokines while suppressing production of proinflammatory cytokines and formation of the M1 phenotype. 

*(3) The Cascade Activation of the Reprogramming Mechanisms Phenomenon.* This phenomenon provides the rapid formation of the desired macrophage phenotype. 

*(4) The Feedback Phenomenon.* Positive feedback provides rapid formation of the desired macrophage phenotype. For instance, the M1 phenotype is produced if there is a need to kill a virus, bacteria, or a tumor cell. Negative feedback prevents excessive M1 phenotype formation, which might result in excessive inflammation followed by the development of inflammatory diseases.

The occurrence of these phenomena is triggered by various intracellular signalling pathways.

### 2.3. The Reprogramming Signaling Pathways

The JNK-, PI3K/Akt-, Notch-, JAK/STAT-, TGF-*β*/SMAD-/non-SMAD-, TLR/NF-*κ*B-, and hypoxia-dependent intracellular signalling pathways [37] are involved in macrophage reprogramming.

#### 2.3.1. The JNK-Signalling Pathway in Macrophage Reprogramming

The JNK- (C-Jun N-terminal kinase-) signalling pathway can be activated via growth factor receptors, cytokines receptors, and G-protein-associated receptors. The role of the JNK pathway in macrophage reprogramming has been demonstrated on adipose tissue macrophages (ATMs). ATMs from control mice have the M2 phenotype, whereas ATMs from obese mice have the M1 phenotype [38]. The ATM M1 phenotype contributes to the development of insulin resistance [38].

**Figure 1:**
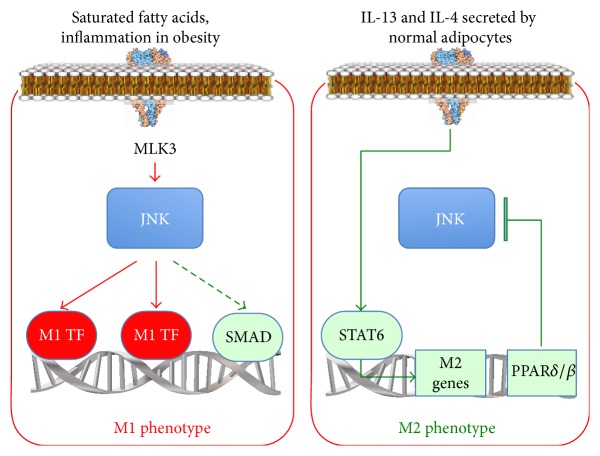
The JNK-signalling pathway in the reprogramming of normal adipose tissue macrophages and those in obesity. TF: transcription factor.

In obesity, saturated fatty acids trigger JNK via mixed-lineage kinase 3 (MLK3) activation. In turn, JNK activates proinflammatory gene expression and thereby reprograms macrophages to the M1 phenotype [37] (Figure 1). However, JNK can also activate the M2 phenotype transcription factor SMAD3 [39]. This suggests that under certain circumstances JNK can be involved in M2 phenotype formation.

Maintaining the ATM M2 phenotype in a lean organism is associated with the fact that normal adipocytes produce RF-М2, such as IL-13 and IL-4. These cytokines activate the macrophage signal transducers and activators of transcription factor-6 (STAT6) which activates the M2 phenotype and macrophage peroxisome proliferator-activated receptor gene expression (PPAR*δ*/*β*). PPAR*δ*/*β* blocks the JNK-dependent reprogramming of the ATM M1 phenotype [40] (Figure 1).

Thus, the JNK-dependent signalling pathway

1. controls the macrophage response to growth factors, cytokines, fatty acids, and ligands of the G-protein-associated receptors;
2. is involved in the reprogramming of macrophages to the M1 phenotype;
3. can activate the M2 phenotype transcription factor SMAD3, thus restricting M1 phenotype formation.

#### 2.3.2. The PI3K/Akt-Signalling Pathway in Macrophage Reprogramming

**Figure 2:**
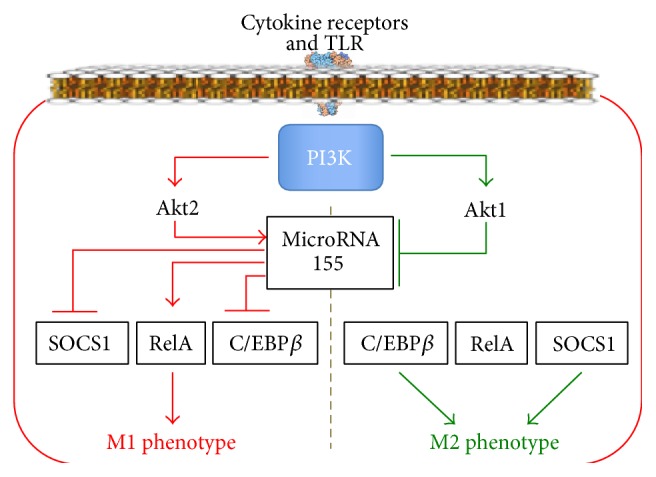
The PI3K/Akt-signalling pathway in macrophage reprogramming.

Phosphatidylinositol-3-kinase (PI3K) is activated via cytokine receptors and TLR. PI3K produces phosphatidylinositol-3,4,5-triphosphate (PIP3), which activates protein kinase Akt. Akt has three isoforms: Akt1, Akt2, and Akt3; Akt1 promotes M2 phenotype formation, and Akt2 promotes M1 phenotype formation [40, 41] (Figure 2). MicroRNA-155 (miR-155) and CAAT/enhancer-binding proteins *β* (C/EBP*β*) play a key role in Akt-dependent macrophage reprogramming [41, 42]. Akt2 enhances the expression of miR-155, which activates RelA/NF-*κ*B transcription factor and inhibits suppressor of cytokine signalling 1 (SOCS1). As a result, Akt2 increases expression of the M1 phenotype genes* iNOS* and* TNF-α*. On the contrary, Akt1 inhibits miR-155, resulting in an increase in C/EBP*β* and the M2 phenotype gene (Arg1 and IL-4) expression [43]. Switching the translation signal between Akt1 and Akt2 seems to be one of the mechanisms responsible for high macrophage plasticity. Arranz et al. [41] and Androulidaki et al. [42] have shown that LPS activates Akt1, thereby enhancing production of anti-inflammatory mediators.

Thus, the PI3K/Akt-dependent signalling pathway

1. mediates the macrophage response to cytokines and ligands of TLR;
2. promotes the reprogramming of macrophages to the M1 phenotype via Akt2 and to the M2 phenotype via Akt1;
3. can induce formation of such a macrophage phenotype that will produce anti-inflammatory mediators in response to LPS.

#### 2.3.3. The Notch-Signalling Pathway in Macrophage Reprogramming

**Figure 3:**
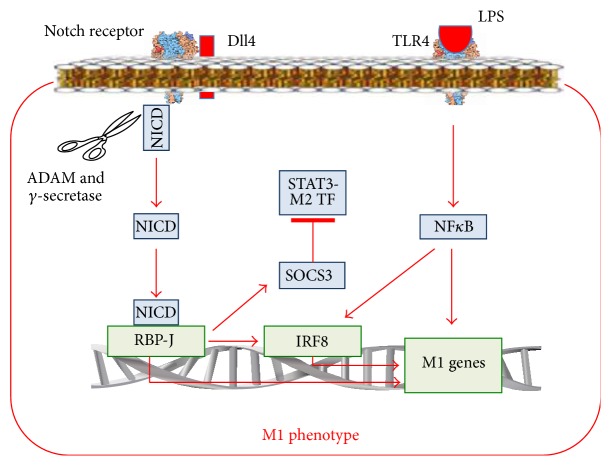
The Notch-signalling pathway in macrophage reprogramming and its interaction with the TLR-dependent signalling pathway. M2 TF: transcription factor of the genes involved in the M2 phenotype formation.

In mammals, there are four transmembrane Notch receptors (Notch-1, Notch-2, Notch-3, and Notch-4) and five transmembrane ligands referred to as Delta-like 1 (Dll1), Dll3, Dll4, Jagged-1, and Jagged-2 [44]. The ligand proteins induce ADAM-proteinase- and *γ*-secretase-mediated splitting of the Notch protein and release of the NICD domain. NICD enters the cell nucleus, binds to RBP-J, and modifies gene expression (Figure 3) [45].

It has been shown that LPS increases the Dll4 content on the surface of macrophages via the TLR4/NF-*κ*B-dependent pathway [46]. Incubation of intact macrophages with macrophages expressing Dll4 induced Notch proteolysis in the intact macrophages (Figure 3). This led to increased activity of genes such as IL-12 and* i*NOS, activation of Akt- and NF-*κ*B-signalling pathways, and the reprogramming of macrophages to the M1 phenotype. Activation of the Notch-signalling pathway provides rapid reprogramming to the M1 macrophage phenotype and is an example of cascade activation of the macrophage reprogramming pathways, that is, from the TLR4/NF-*κ*B-dependent pathway to the Notch-signalling pathway.

Xu et al. [45] have shown that Notch1 activation enhances RBP-J formation. RBP-J increases the expression of interferon regulatory factor 8 (IRF8) transcription factor in macrophages. In turn, IRF8 contributes to the activation of inflammatory genes, such as IL-12 [47]. IRF8 is involved not only in the IFN-*γ* and Notch pathways, but also in the TLR-4-signaling pathway of activation of proinflammatory M1 cytokines [48]. Therefore, the binding of ligands to TLR4 on reprogrammed macrophages with an increased content of IRF8 will lead to a more prominent inflammatory response compared to that of macrophages which were not reprogrammed. This could explain the mechanism of an enhanced proinflammatory M1 response of macrophages after the reprogramming, based on the convergence of the Notch1-RBP-J-, IFN-*γ*-, and TLR4-signaling pathways at the level of IRF8 (Figure 3).

Wang et al. [49] have shown that expression of SOCS3, which is an inhibitor of the M2 transcription factor of STAT3 genes, increases in Notch/RBP-J-dependent reprogramming of macrophages to the M1 phenotype (Figure 3). These findings could explain the mechanism linking an increase in proinflammatory cytokines production with a decrease in anti-inflammatory cytokines production.

Other investigators, using the macrophages from mice with systemic lupus erythematosus, have shown that the Notch1-signaling pathway can be involved also in the formation of the pathogenic M2 phenotype [50]. In this case, the Notch1 pathway translates the signal via the PI3K and MAPK pathways and accelerates the translocation of NF-*κ*B p50 to the nucleus. As the dimer p50/p50 activates genes of the M2 phenotype [51], the Notch1/NF-*κ*B p50 pathway reprograms macrophages to the M2 phenotype.

Thus, the Notch-dependent signalling pathway

1. mediates the reaction of macrophages to Delta-like and JAG ligands and, indirectly, to TLR4 ligands LPS;
2. reprograms the M1 macrophage phenotype via NICD, RBP-J, and IRF8 activation;
3. in systemic lupus erythematosus seems to be able to participate in the M2 phenotype formation via the activation of PI3K/MAPК/NF-*κ*B p50 pathways;
4. can participate in key macrophage reprogramming phenomena: (a) by enhancing the response of the M1 macrophages to proinflammatory stimuli due to the convergence of Notch1-RBP-J, IFN-*γ*, and TLR4 pathways at the level of IRF8, (b) by linking an increase in production of proinflammatory cytokines with a decrease in production of anti-inflammatory cytokines since Notch/RBP-J-dependent reprogramming to the M1 phenotype increases expression of inhibitor of M2 transcription factor of STAT3 genes (SOCS3), and (c) by cascade activation of the reprogramming pathways, due to the fact that the TLR4/NF-*κ*B pathway increases the amount of Dll4, ligands of the Notch pathway.

#### 2.3.4. The JAK/STAT-Signalling Pathway in Macrophage Reprogramming

**Figure 4:**
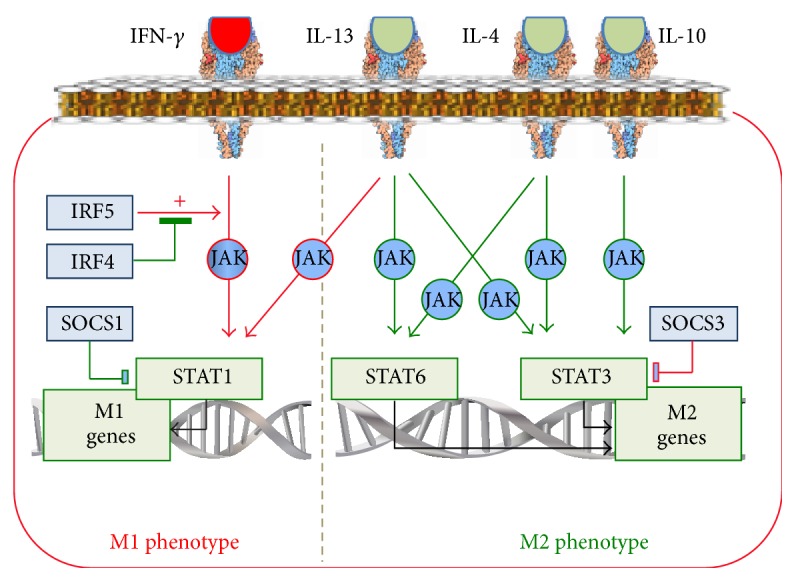
The JAK/STAT-signalling pathway in macrophage reprogramming.

In immune cells, the JAK/STAT-signalling pathway transmits signals from IFN-*γ*, IL-2, IL-4, IL-7, IL-9, IL-13, IL-15, and IL-21 via cytokines receptors [52]. The JAK/STAT pathway employs four JAK (just another kinase or Janus kinase), namely, JAK1, JAK2, JAK3, and Tyk2, plus 7 transcription factors, STAT, namely, STAT1–4, STAT5A, STAT5B, and STAT6 [53]. The receptors of the JAK/STAT pathway are activated by RF-M1, for example, IFN-*γ*, as well as by RF-M2, for example, IL-4 (Figure 4) [53].

IFN-*γ* via the JAK/STAT pathway induces STAT1 activation, which results in an increase in the production of proinflammatory cytokines and, therefore, programs macrophages to the M1 phenotype [54] (Figure 4). The IFN-*γ*/JAK/STAT1 pathway is under control of IRF5 and IRF4 [55]. IRF5 is activated by proinflammatory factors [56], whereas IRF4 is activated by anti-inflammatory factors [57]. IRF5 enhances the IFN-*γ*/JAK/STAT1-dependent production of the M1 cytokine IL-12 [58], whereas IRF4 suppresses the effect of IRF5 [57]. Thus, IRF4 and IRF5 exert opposite effect on the IFN-*γ*/JAK/STAT1 pathway and on the macrophage phenotype.

The IFN-*γ*/JAK/STAT1 pathway is involved in the increase of the macrophage response to TLR ligands after preconditioning with IFN-*γ*. The basis of this phenomenon is the convergence of the IFN-*γ*/JAK- and TLR4-signaling pathways at the level of STAT1 [59]. Therefore, the increased activity of STAT1 in the IFN-*γ*-reprogrammed macrophages produces a more intensive response to TLR4 ligands.

IL-4, IL-13, and IL-10 reprogram macrophages to the M2 phenotype (Figure 4) via the JAK/STAT-signalling pathways. The binding of IL-4 with its receptor activates JAK. Subsequently, however, in contrast to IFN-*γ*, the phosphorylation and activation of the transcription factors of the M2 phenotype gene, STAT3 and STAT6, occur [60]. In addition, IL-4 induces expression of c-Myc, which increases the expression of the M2 phenotype genes, such as Scarb1 and Mrc1, and the activity of STAT6 and PPAR-*γ* [61].

Binding of IL-13 with its receptor activates JAK1, JAK2, and Tyk2 kinases with subsequent activation of STAT1, STAT3, and STAT6 [60]. After that, STAT3 and STAT6 activate expression of M2 phenotype genes, such as mannose receptor, Fizz1, Ym1, and anti-inflammatory cytokines [19], whereas STAT1 activates proinflammatory cytokines (Figure 4). The macrophage anti-inflammatory response to IL-13 seems to be predetermined by the prevalence of the activation of STAT3 and STAT6 over STAT1. However, it could be suggested that the blocking of STAT3 and STAT6 might lead to the formation of such a macrophage phenotype which could produce proinflammatory cytokines in response to the anti-inflammatory cytokine IL-13.

Binding of IL-10 with its receptor via activation of JAK1 and STAT3 induces expression of the M2 phenotype genes, such as TGF-*β* and IL-10 [19], as well as the genes which inhibit production of M1 cytokines, such as TNF-*α* [62]. In addition, IL-10 stimulates the relocation of p50/p50 into the nucleus, where it blocks the proinflammatory gene expression [63].

There are two important regulators of cross M1/M2 macrophage reprogramming inherent to the JAK/STAT-signalling pathway. These are the SOCS1 and SOCS3 proteins [64]. IL-4 activates the synthesis of SOCS1, which blocks STAT1, thereby preventing the M1 phenotype formation. IFN-*γ* and the TLR4 ligands activate the synthesis of SOCS3, which blocks STAT3, thereby preventing formation of the M2 phenotype [65] (Figure 4). Concurrently, SOCS1 activates STAT6, the M2 phenotype transcription factor, while SOCS3 activates STAT1, the M1 phenotype transcription factor. Such interactions between SOCS and STAT provide a further explanation of the link between the rise in the production of proinflammatory cytokines and the decrease in the production of anti-inflammatory cytokines during the reprogramming to the M1 phenotype, and vice versa.

Thus, the JAK/STAT-dependent signalling pathway

1. mediates the response of the macrophages to IL-2, IL-4, IL-7, IL-9, IL-13, IL-15, IL-21, and IFN-*γ*;
2. translates signals into the nucleus from the M1 cytokine IFN-*γ* and, via STAT1, reprograms macrophages to the M1 phenotype; this pathway also translates signals from the M2 cytokines, for example, IL-4, IL-10, and IL-13, and via STAT3 and STAT6 reprograms macrophages to the M2 phenotype; this allows the macrophage to integrate the reprogramming effect of the microenvironment containing different cytokines;
3. triggered from the IL-13 receptors transmits signals to the M1-reprogramming STAT1 and the M2-reprogramming STAT3 and STAT6; due to this, the JAK/STAT signalling pathway seems to be able under the M2-reprogramming pathways suppression to form a phenotype, which in response to RF-M2 IL-13 will increase the M1 cytokines production and form the M1 phenotype;
4. participates in the phenomenon of the augmentation of the macrophage proinflammatory response to different inflammatory stimuli after the macrophage reprogramming due to the IFN-*γ*/JAK and TLR4 pathway convergence at the level of STAT1;
5. participates in the phenomenon of the link between the rise in the proinflammatory cytokines production and the decrease in the anti-inflammatory cytokines production during the reprogramming of macrophages to the M1 phenotype, and vice versa, due to the SOCS and STAT interactions.

#### 2.3.5. The TGF-*β*-Signalling Pathway in Macrophage Reprogramming

**Figure 5:**
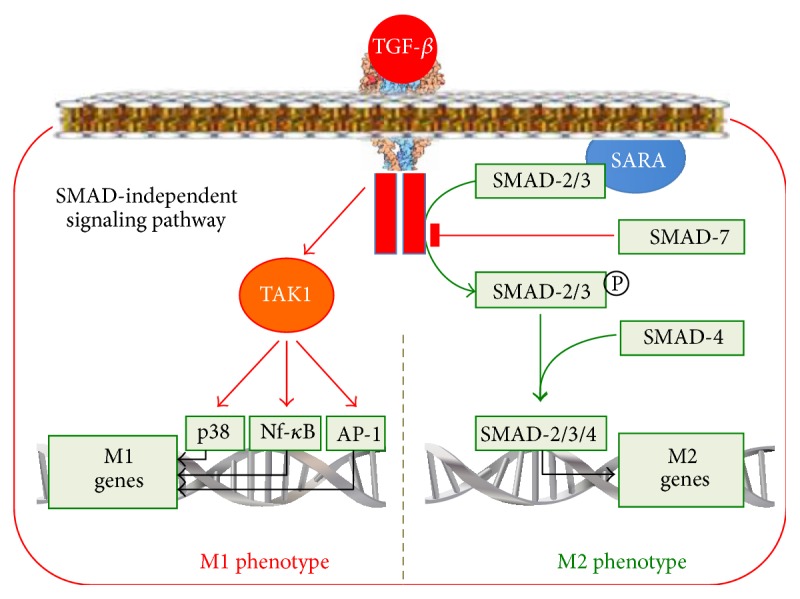
The TGF-*β*-signalling pathway in macrophage reprogramming.

The family of secretory TGF-*β* proteins includes TGF-*β*1, TGF-*β*2, TGF-*β*3, activins, and growth factors. The immune cells mostly produce TGF-*β*1. TGF-*β* receptor consists of two transmembrane subunits of type I (T*β*RI) and two subunits of type II (T*β*RII) with the cytoplasmic kinase domains. After TGF-*β* binding to its receptor, the T*β*RII subunit autophosphorylates and phosphorylates T*β*RI. As a result, the T*β*RI kinase domain binds to the SMAD2 and SMAD3 transcription factors (Figure 5). The SMAD Anchor for Receptor Activation (SARA) protein helps to attract SMAD2/3 to T*β*RI [66]. After that, T*β*RI phosphorylates and activates SMAD2/3. The activated SMAD2/3 binds to SMAD4, and the SMAD2/3/4 complex is translocated into the nucleus.

The TGF-*β*-activated, SMAD-dependent pathways upregulate the activity of the M2 phenotype genes, such as arg1 and mgl2 [67], and reprogram the macrophage to the M2 phenotype. The TGF-*β*-dependent reprogramming to the M2 phenotype is under the SMAD7 control. SMAD7 can bind to T*β*RI, thereby preventing SMAD2/3 phosphorylation, or it can direct SMAD2/3 to the proteasomes for degradation. IFN-*γ* and TNF-*α* can upregulate the SMAD7 expression leading to the inhibition of the TGF-*β*/SMAD pathway and the decrease in the anti-inflammatory cytokines production in response to TGF-*β*. This mechanism leads to understanding of the link between the increase in proinflammatory cytokines production and the decrease in anti-inflammatory cytokines production.

In addition to the SMAD-dependent pathway, TGF-*β* can activate SMAD-independent pathways, including Ras/MAPK/Erk, PI3K/Akt, p38, and JNK as well as Rho-like GTPases [39] pathways. In SMAD-independent pathways, the TGF-*β* activated kinase 1 protein (TAK1) transduces the signal from TGF-*β* to several downstream signalling cascades, including MAP kinase pathways, JNK, p38, and NF-*κ*B [68] (Figure 5). It can be suggested that SMAD-independent pathways, which activate proinflammatory proteins and transcription factors such as JNK, p38, and NF-*κ*B, are able to reprogram macrophages to the M1 phenotype, particularly when the SMAD-dependent pathway is blocked.

Thus, we observe the following in the TGF-*β*-dependent signalling pathway:

1. It mediates the reaction of macrophages to TGF-*β* ligands.
2. It translates the signal from TGF-*β* via the SMAD2/3/4-dependent pathway, thereby reprogramming macrophages to the M2 phenotype.
3. TGF-*β* seems to be able to form the macrophage phenotype, which in case of the suppression of SMAD-dependent pathways will increase production of the M1 cytokines and form the M1 phenotype in response to RF-М2 TGF-*β* via the SMAD-independent TAK1/JNK/p38/NF-*κ*B-dependent pathways.
4. It contributes to the link between an increase in proinflammatory cytokines production and a decrease in anti-inflammatory cytokines production in the M1 phenotype. This results from the fact that the IFN-*γ*- or TNF-*α*-mediated macrophage reprogramming to the M1 phenotype increases the expression of SMAD7, which blocks SMAD2/3/4 formation and anti-inflammatory M2 cytokines production.

#### 2.3.6. The TLR/NF-*κ*B-Signalling Pathway in Macrophage Reprogramming

**Figure 6:**
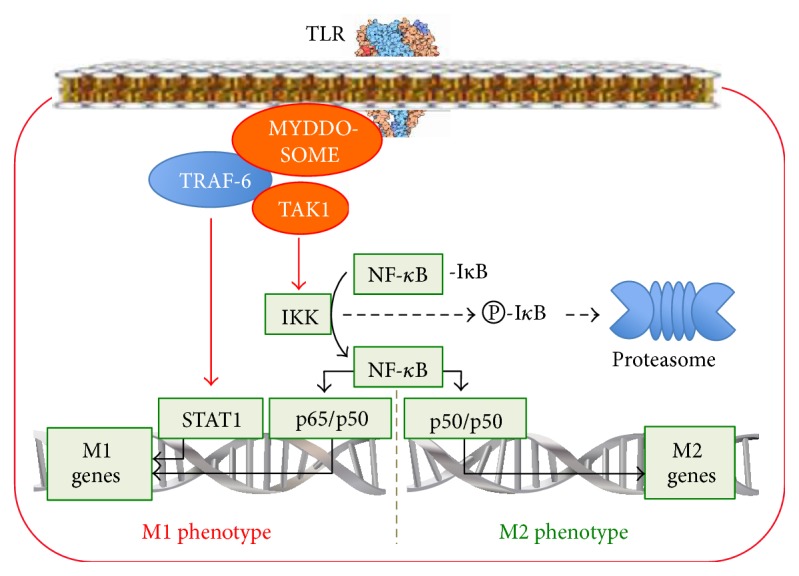
The TLR/NF-*κ*B-signalling pathway in macrophage reprogramming.

Toll-like receptors (TLR) belong to the family of transmembrane PRR, which recognize specific PAMP on the molecules of microbes. The binding of PAMP to TLR triggers the signalling cascades, which induce the expression of proinflammatory cytokines. Six TLR (TLR1, TLR2, TLR4, TLR5, TLR6, and TLR10) have been identified on the surface of macrophages. Each TLR consists of ligand-sensitive, transmembrane, and TIR domains [69]. When a ligand binds to a TLR, it forms a dimer. As a result, the TIR domains become closer to each other and start to interact with myeloid differentiation primary-response gene 88 (MyD88) [70]. MyD88 binds to the members of the IL-1R associated kinase (IRAK) family leading to the formation of the oligomeric Myddosome complex [71] (Figure 6). Phosphorylation of IRAK occurs in the Myddosome complex. Phosphorylated IRAK attract tumor necrosis factor receptor-associated factor-6 (TRAF6) to the membrane [72]. In turn, TRAF6 attracts the TAK1 complex.

TAK transduces the signal from TLR and the TGF-*β*, TNF-*α*, and IL-1 receptors [68]. There is the convergence of the signals from pro- (TNF-*α*) and anti-inflammatory (TGF-*β*) cytokines at the level of TAK. Due to this, macrophages are probably able to respond to proinflammatory factors (RF-M1), for instance, TNF-*α*, by producing anti-inflammatory cytokines and, conversely, by producing proinflammatory cytokines in response to anti-inflammatory factors (RF-M2) such as TGF-*β*. Obviously, in the first case, this mechanism prevents the development of excessive inflammation, whereas in the second case a critical decrease in bactericidal, antiviral, and antitumoral properties of macrophages occurs during the Th2 response development. In both cases, it could be concluded that TAK can form a special macrophage phenotype.

The attraction of TAK complexes to TRAF6 is accompanied by the convergence of the TAK kinase domains. This causes the autophosphorylation and activation of TAK1-kinase, which, in turn, activates the complex containing I*κ*B-kinase (IKK) [73] (Figure 6). IKK phosphorylates the inhibitory subunit I*κ*B, which is associated with the NF-*κ*B transcription factor in the cytoplasm of inactive macrophages. Phosphorylation of I*κ*B leads to its degradation in proteasomes. As a result, free NF-*κ*B is transported into the nucleus, where it activates the genes involved in inflammation, immune responses, and cellular growth [74]. The proteins belonging to the NF-*κ*B RelA (p65), RelB, and c-Rel families can activate the expression of proinflammatory genes whereas those belonging to the p50, p52 Relish families cannot. Proinflammatory cytokines produced by the NF-*κ*B-dependent pathway can repeatedly activate the NF-*κ*B-dependent pathway. This is how a positive feedback is formed, which provides rapid formation of the proinflammatory phenotype. In addition, NF-*κ*B activates the I*κ*B genes. This mechanism restricts the excessive transportation of NF-*κ*B into the nucleus and represents a negative feedback, which prevents the development of an excessive inflammation.

In most cases, the TLR/NF-*κ*B-signalling pathway reprograms macrophages to the M1 phenotype in response to microbial invasion. There are three interesting and important features of TLR/NF-*κ*B-dependent reprogramming.

Firstly, during LPS-dependent macrophage reprogramming to the M1 phenotype, the NF-*κ*B activation occurs in the form of p65/p50, which enhances the production of proinflammatory cytokines. Simultaneously, LPS enhances the expression of the nuclear I*κ*B*ζ* genes [75]. I*κ*B*ζ* blocks the p50/p50 form of NF-*κ*B, which stimulates the production of anti-inflammatory cytokines [76]. This can serve as another mechanism of the link between an increase in the proinflammatory cytokines production and a decrease in the anti-inflammatory cytokines production in the M1 phenotype.

Secondly, the TLR/NF-*κ*B pathway can switch the phenotype reprogramming due to a change in the NF-*κ*B subunit composition. If the activation of NF-*κ*B occurs in the form of p50/p65, then the production of proinflammatory cytokines increases [77], and the M1 phenotype is formed. If the activation of NF-*κ*B occurs in the form of p50/p50, then the M2 phenotype is formed as it occurs in tumor-associated and LPS-tolerant macrophages [76]. This is one of the mechanisms regulating the macrophage plasticity, which allows for a rapid response to changes in infection and microenvironment. The possibility to switch between the M2 genes transcription factor p50/p50 [76] and the M1 genes transcription factor p50/p65 [77] in response to the action of the same ligand, LPS, suggests the possibility of forming a macrophage phenotype that will produce anti-inflammatory factors and, thereby, manifest itself as the M2 phenotype in response to RF-M1 (LPS).

Thirdly, it has been shown that the binding of LPS to TLR4 via TRAF6 induces phosphorylation of the STAT1 transcription factor [78]. After its activation, STAT1 is translocated into the nucleus where it activates proinflammatory genes, such as TNF-*α*. These data show the possibility of switching between different pathways, namely, from the TLR- to the STAT1-dependent pathway. This mechanism reflects the phenomenon of cascade activation of macrophage reprogramming pathways and might well contribute to the phenomenon of the increased production of proinflammatory cytokines after the reprogramming of macrophages to the M1 phenotype. An example would be the increased production of TNF-*α* in response to the ligands of the JAK/STAT pathway after the TLR-dependent reprogramming.

Thus, we observe the following in the TLR-dependent signalling pathway:

1. It mediates the reprogramming of macrophages in response to ligands of TLR.
2. It activates NF-*κ*B and STAT1 and, thereby, upregulates the production of proinflammatory genes and reprograms macrophages to the M1 phenotype.
3. Due to the possibility of producing NF-*κ*B in two different forms such as p65/p50, which is the proinflammatory M1 form, or p50/p50, which is the anti-inflammatory M2 form, the TLR-dependent pathway seems to be able to program the macrophage phenotype, which will then increase the production of the M2 cytokines in response to RF-M1 such as LPS.
4. It contributes to the phenomenon of reprogramming: (a) contributes to the link between an increase in the proinflammatory cytokines production and a decrease in the anti-inflammatory cytokines production in the M1 phenotype (the reprogramming of macrophages to the M1 phenotype due to LPS upregulates the expression of the nuclear I*κ*B*ζ*, which blocks the p50/p50 anti-inflammatory form of NF-*κ*B); (b) contributes to the increase in the production of proinflammatory cytokines in response to the ligands of the JAK/STAT pathway after TLR-dependent macrophage reprogramming to the M1 phenotype (the phenomenon is based on the TLR-dependent activation of STAT1, which is a transcription factor of the JAK/STAT pathway); (c) contributes to the cascade activation of the macrophage reprogramming pathways as a result of signal switching from the TLR-dependent pathway to the STAT1-dependent pathway via TRAF6; (d) contributes to positive and negative feedback due to proinflammatory cytokines, which can repeatedly activate NF-*κ*B, and due to NF-*κ*B-dependent synthesis of the inhibitor I*κ*B, respectively.

#### 2.3.7. The Hypoxia-Dependent Pathway of Macrophage Reprogramming

The intracellular protein complex of the oxygen sensor [79] consisting of the prolyl-4-hydroxylase domain (PHD) enzyme and the hypoxia-inducible transcription factor (HIF) plays a key role in the hypoxic reprogramming of the macrophage phenotype. HIF consists of two subunits: a constitutive HIF-*β* subunit and an oxygen-sensitive subunit, HIF-1*α*, HIF-2*α*, or HIF-3*α*. Under normoxic conditions, HIF-*β* is constantly present in the cell, whereas HIF-*α* is constantly synthesized but not accumulated because of its PHD-mediated binding to the hydroxyl group [80] with subsequent relocation to proteasomes and degradation therein.

Under hypoxia, the PHD activity decreases and, as a result, HIF-*α* accumulates. Then HIF-*α* binds to HIF-*β* and forms the HIF-*α*/HIF-*β* dimer. This dimer penetrates the nucleus and activates the gene [79], which increase the endurance of macrophages to hypoxia. Either HIF-1*α* or HIF-2*α* is activated in macrophages depending on the extent of the hypoxia. HIF-1*α* induces* i*NOS synthesis and promotes the M1 phenotype formation, whereas HIF-2*α* activates arginase 1 and promotes the M2 phenotype formation [24].

#### 2.3.8. The Conclusions regarding the Signalling Mechanisms of Macrophage Reprogramming

**Figure 7:**
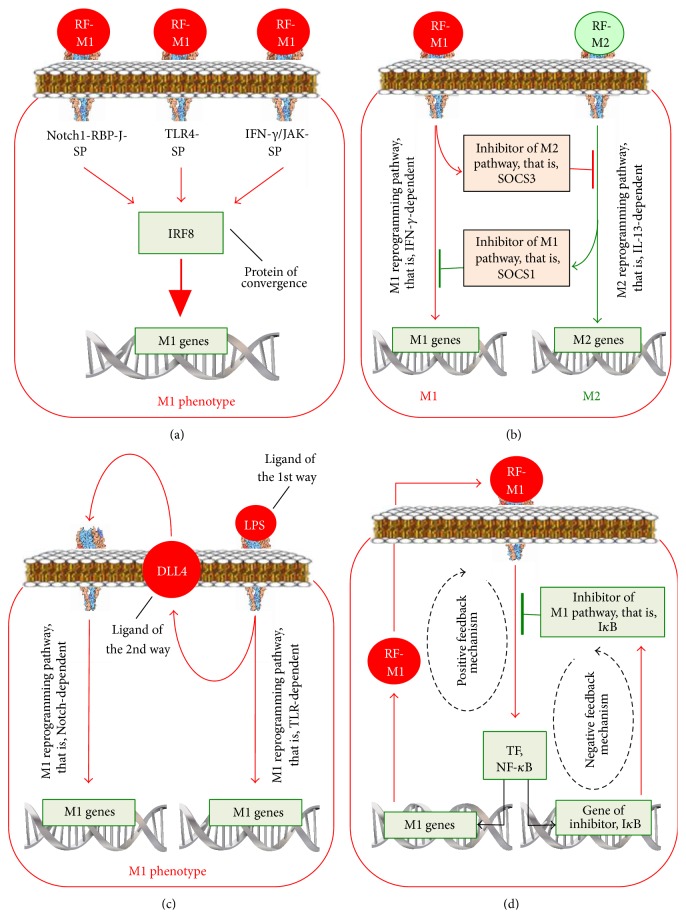
The main phenomena of macrophage reprogramming. (a) The phenomenon of an amplified macrophage response to both the reprogramming factor (direct amplification) and another factor (cross amplification). (b) The phenomenon of reciprocal suppression of the alternate phenotype. (c) The phenomenon of a cascade activation of the reprogramming mechanisms. (d) The feedback phenomenon.

1. There is a relative specialization of the signalling pathways of macrophage reprogramming in response to different components of the microenvironment. In particular, growth factors, fatty acids, and the ligands of G-protein-associated receptors reprogram macrophages via the JNK-dependent pathway; microbial invasion, namely, PAMP via the TLR-, PI3K/Akt-, and Notch-dependent pathways; changes in the Delta-like and JAG ligand content in the microenvironment via the Notch-dependent pathways; changes in the cytokine microenvironment via the JNK-, PI3K/Akt-, and JAK/STAT-dependent signalling pathways; changes in the TGF-*β* content via the SMAD-dependent and SMAD-independent signalling pathways; and changes in the oxygen content via the hypoxia/HIF-dependent pathways.
2. There are two types of macrophage reprogramming signalling pathways: pathways which mainly program the M1 phenotype such as JNK, Notch, TLR/NF-*κ*B (р65/р50), PI3K/Akt2, JAK/STAT1, and HIF1*α* pathways and those pathways which mainly program the M2 phenotype such as PI3K/Akt1, JAK/STAT3/6, TGF-*β*/SMAD, TLR/NF-*κ*B (р50/р50), and HIF2*α* pathways.
3. An understanding of the reprogramming mechanisms allows us to explain the four main phenomena observed during the course of macrophage reprogramming: (1) the phenomenon of an amplified macrophage response to both the reprogramming factor (direct amplification) and another factor (cross amplification), (2) the phenomenon of reciprocal suppression of the alternate phenotype, (3) the cascade activation of the reprogramming mechanisms phenomenon, and (4) the feedback phenomenon.
4. The enhanced macrophage response to the factor with which the macrophages have been reprogrammed (direct amplification) or their response to another factor (cross amplification) is based on the convergence of signalling pathways (Figure 7(a)) at the level of a certain protein. Therefore, macrophage reprogramming via a particular signalling pathway that leads to an increased activity or content of this protein results in an increase in the subsequent response of the macrophages to the ligands of another pathway. IRF8 is the convergence protein for the M1-reprogramming Notch1-RBP-J, IFN-*γ*, and TLR4 pathways. STAT1 is the convergence protein for the IFN-*γ*/JAK and TLR4 pathways.
5. The phenomenon of reciprocal suppression of an alternative phenotype is caused by the fact that the formation of a phenotype is accompanied by an increase in synthesis of the molecules suppressing the formation of the alternative phenotype (Figure 7(b)). In particular, the M1 phenotype formation via (1) the Notch/RBP-J, IFN-*γ*, or TLR4 pathway is accompanied by an increase in the expression of SOCS3, which suppresses the activity of the M2 genes transcription factor STAT3; (2) the IFN-*γ* and TNF-*α* pathways by an increase in the expression of SMAD7 blocks the activation of the M2 genes transcription factors SMAD2/3/4; (3) the TLR/NF-*κ*B (p65/p50) pathways increases the expression of inhibitors of the M2 genes. The M2 phenotype formation via the IL-4/JAK/STAT pathway is accompanied by an increase in SOCS1 synthesis, which blocks the M1 genes transcription factor STAT1.
6. The phenomenon of cascade activation of the reprogramming pathways is based on the ability of one reprogramming pathway to transmit the signal to another pathway. In particular, the LPS/TLR4/NF-*κ*B pathway of the M1 reprogramming increases the amount of Dll4, which is a ligand of the Notch pathway of the M1 reprogramming, whereas the TLR-dependent pathway via TRAF6 transmits the signal to the STAT1-dependent pathway (Figure 7(c)).
7. The phenomenon of positive and negative feedback is based on the ability of a signalling pathway to upregulate both its own activators and inhibitors. In particular, the NF-*κ*B-dependent pathway of the M1 reprogramming upregulates the amount of both proinflammatory cytokines, which activate this pathway, thereby forming positive feedback, and I*κ*B, which inhibits NF-*κ*B, thereby forming negative feedback (Figure 7(d)).
8. These phenomena, on the one hand, provide rapid formation of M1 macrophages if it is necessary to eliminate bacteria or viruses or formation of M2 macrophages if there is a need to kill parasites, enhance angiogenesis, or repair tissue. At the same time, these phenomena prevent excessive activation of the phenotype.
9. The signalling pathways that program the proinflammatory M1 macrophage phenotype often have an offshoot, which, if activated, can upregulate the production of the anti-inflammatory M2 cytokines, and vice versa. In particular, the JNK-dependent pathways activate the M1 phenotype transcription factors, but they can also activate the M2 genes transcription factor SMAD3. The PI3K/Akt-dependent pathway programs the M1 phenotype via Akt2 and the M2 phenotype via Akt1. The Notch pathway increases the production of the M1 cytokines via NICD/RBP-J/IRF8 and the production of M2 cytokines via PI3K/MAPК/NF-*κ*B p50. IL-13 programs the M2 phenotype via JAK2/STAT3 and Tyk2/STAT6 pathways and the M1 phenotype via Tyk2/STAT1 pathway. TGF-*β* programs the M2 phenotype via SMAD2/3/4 and the M1 phenotype via TAK1/JNK/p38/NF-*κ*B. Lastly, the TLR-dependent pathway programs the M1 phenotype via p65/p50 and the M2 phenotype via p50/p50.
10. These signalling intracellular pathways play a crucial but not the sole role in macrophage reprogramming. They can activate or inhibit the mechanisms of other levels of cell activity and cell phenotype regulation, namely, posttranscriptional ones.

### 2.4. The Posttranscriptional Mechanisms of Macrophage Reprogramming

The key element of posttranscriptional regulation of many cellular processes, including macrophage reprogramming, is microRNA (miR) [81]. miRs are small, noncoding molecules of RNA comprised of 18–25 nucleotides. miRs bind with mRNA and inhibit translation of a protein [82]. One miR can control several mRNA. In addition, miR can interact with DNA during the course of methylation of DNA that suppresses activity of genes [83].

#### 2.4.1. RF-M1s, Specific Sets of miRs, and Their Effects on the Phenotype of Macrophages

**Figure 8:**
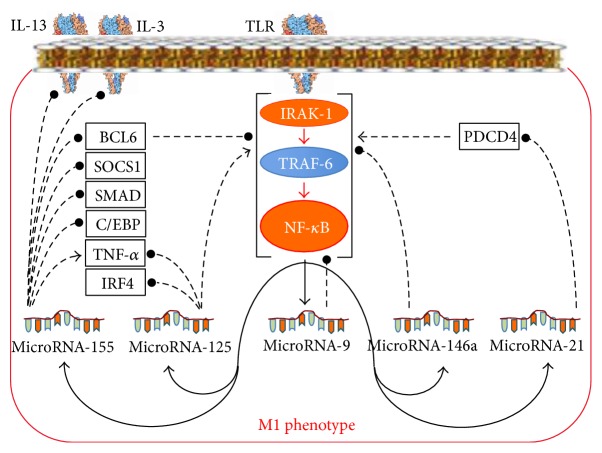
A specific set of miRs whose synthesis is increased in response to the M1 reprogramming factors and miR-dependent mechanisms of the M1 phenotype formation. Lines with arrowheads: activating influences; lines with filled dot heads: inhibiting influences.

**Figure 9:**
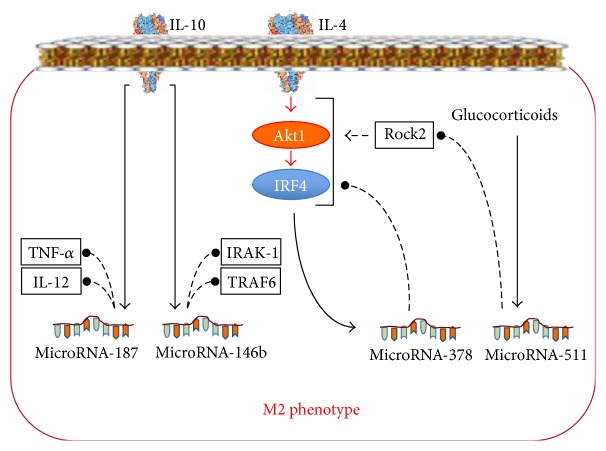
A specific set of miRs whose synthesis is increased in response to the M2 reprogramming factors and miR-dependent mechanisms of the M2 phenotype formation. Lines with arrowheads: activating influences; lines with filled dot heads: inhibiting influences.

In macrophages, factors of macrophage reprogramming activate the synthesis of specific sets of miRs that control balance of pro- and anti-inflammatory processes. In particular, RF-M1s induce the synthesis of miR-155, miR-21, miR-125, miR-9, and miR-146 (Figure 8) via TLR, whereas RF-M2s induce the synthesis of miR-511 via the glucocorticoid receptor, the synthesis of miR-187 via the IL-10 receptor, and the synthesis of miR-378 via the IL-4 receptor [17, 84] (Figure 9).

Among all miRs, the synthesis of which increases under the influence of ligands of TLR, miR-155 has been the most investigated (Figure 8). miR-155 increases stability of the TNF-*α* transcript [85], decreases synthesis of SOCS1, and, thereby, promotes the development of antiviral immunity [86]. It blocks IL-13 and IL-3 receptors [87, 88], blocks SMAD2, and, thereby, decreases the M2-reprogramming effect of TGF-*β* [89]. miR-155 also blocks the translation of transcription factor BCL6 and, as a result, promotes the development of atherosclerosis [90]. In addition, miR-155 blocks transcription factor C/EBP-*β* and, thereby, decreases production of cytokine IL-10 [91], which is RF-M2. miR-155 can also shift the M2 phenotype of TAM towards the M1 phenotype and, thereby, enhance antitumoral properties of macrophages [92].

miR-29b and miR-125, induced via TLR, increase proinflammatory NF-*κ*B signalling [93, 94] and block the translation of signal from the IL-4 receptor at the level of IRF4 [95] (Figure 8). These miRs also promote the formation of the M1 phenotype. At the same time, miR-125a and miR-125b can decrease stability of the TNF-*α* transcript [96].

miR-146a [97], miR-9 [98], miR-21 [99], and miR-147 [100], induced in macrophages via TLR, prevent M1 phenotype reprogramming (Figure 8). miR-146a blocks IRAK-1 and TRAF6 in the TLR-signalling pathway [97]. Due to this action miR-146a plays an important role in the formation of LPS-tolerant macrophages [101]. miR-9 suppresses the proinflammatory activity of NF-*κ*B directly [98], whereas miR-21 indirectly suppresses this activity via PDCD4 [99].

Thus, the miRs induced via TLR form a regulatory network, in which two functional contours can be distinctly differentiated (Figure 8): (1) the positive feedback mechanism, which is formed by miR-155 and miR-125, which provides rapid formation of the M1 phenotype to eliminate an infection, and (2) the negative feedback mechanism, formed by miR-146a, miR-9, miR-21, and miR-147, which seems to prevent excessive inflammation in the immune response to an infection.

#### 2.4.2. RF-M2, Specific Sets of miRs, and Their Effects on the Phenotype of Macrophages

Similar to RF-M1, RF-M2 induces another specific set of miRs in macrophages (Figure 9). IL-10 increases the synthesis of miR-187, which promotes the termination of inflammation by blocking the transcripts of TNF-*α* and IL-12 [102]. IL-10 also increases the synthesis of miR-146b, which suppresses the production of proinflammatory cytokines by affecting the key points of the TLR pathway [103]. IL-4 increases the expression of miR-378 [102]. miR-378 is coexpressed together with the M2 gene PPAR-*γ* but acts as an inhibitor of the M2 responses, because it blocks the IL-4/PI3K/Akt-signaling pathway [102]. Glucocorticoids increase synthesis of miR-511 in M2 macrophages. miR-511-3p disturbs the translation of Rock2, which is the factor that promotes the reprogramming of macrophages to the M2 phenotype [104]. Thus, miR-511 and miR-378 are negative regulators of the macrophage M2 response.

Thus, the miRs induced by RF-M2 form a regulatory network, in which, similar to RF-M1, two functional contours can be distinctly differentiated (Figure 9): (1) the positive feedback mechanism, formed by miR-187 and miR-146b, which provides rapid formation of the M2 phenotype to eliminate a parasitic infection or to prevent excessive inflammation induced by viruses and bacteria, and (2) the negative feedback mechanism, formed by miR-378 and miR-511, which limits a significant decrease in the bactericidal and antiviral activity of the immunity during the development of the Th2 response.

#### 2.4.3. Conclusions on the Posttranscriptional Mechanisms of Macrophage Reprogramming

In general, the intracellular network of miRs possesses all the features of a regulatory mechanism of macrophage reprogramming, including both positive and negative feedbacks, and a specificity of action on the receptors and proteins participating in macrophage reprogramming. A few generalities can be made concerning the miR-dependent regulation of the phenotype of macrophages:

1. There is a certain specificity in the expression of miRs; miR-155, miR-21, miR-29b, miR-125, miR-9, miR-146a, and miR-147 are expressed in response to RF-M1, whereas miR-146b, miR-511, miR-187, miR-378, miR-222, miR-27a, miR-125a-3p, and miR-125a-5p are expressed in response to RF-M2.
2. miRs can form mechanisms of positive feedback to provide rapid reprogramming of macrophages to a desired phenotype and of the negative feedback to limit excessive inflammation in case of the M1 phenotype and a significant decrease in the bactericidal, antitumoral, and antiviral activity in case of the M2 phenotype.
3. Understanding of the functions of miRs allows us to explain the mechanisms of macrophage reprogramming.

The role of miRs in the enhanced response of the reprogrammed macrophages is to form a positive feedback, which enhances the response of reprogrammed macrophages. For instance, miR-155, the expression of which is initiated from TLR, blocks the translation of BCL6, which inhibits NF-*κ*B-dependent signalling [93]. With BCL6 blocked, the response of reprogrammed macrophages to the ligands of TLR will be enhanced.

The contribution of miRs to the reciprocal suppression of an alternative macrophage phenotype is determined by the fact that the miR participating in the formation of one or another macrophage phenotype can suppress the signalling pathways producing an alternative phenotype. In particular, miR-155, which participates in the formation of the M1 phenotype, blocks the signalling pathways leading to the M2 phenotype, namely, the receptors of IL-13 [87] and IL-3 [79], SMAD2 [89], and C/EBP-*β* [105].

miRs are also involved in the phenomenon of the cascade activation of the reprogramming pathways. This is due to the fact that the synthesis of many of miRs is increased during the activation of the cytokine-dependent pathways of macrophage reprogramming. For instance, this occurs when IL-10 mediated synthesis of miR-146 increases.

## 3. Paradoxical Plasticity of Macrophages: The M3 Phenotype (Update)

### 3.1. The Current Concept of Macrophage Reprogramming Cannot Explain the Mechanism of the Transition between the M1 and M2 Phenotypes

A disturbance in the reprogramming of macrophages during disease can be associated with an inadequate alteration of a specific phenotype. For instance, inadequate acquiring of the M2 phenotype and excessive production of anti-inflammatory cytokines can decrease bactericidal and antiviral functions of the immune response and provoke allergic and asthmatic Th2 reactions [106, 107] and tumor growth [108]. On the other hand, if there is no change in phenotype when it is necessary, for instance, if phenotype does not change from the M1 to the M2 type at the end of an inflammatory reaction after the elimination of pathogenic microbes, excessive production of the proinflammatory M1 mediators followed by tissue damage and development of proinflammatory diseases can occur [109].

Switching from the M1 to the M2 phenotype protects an organism from excessive inflammation, whereas switching from the M2 to the M1 phenotype protects from allergic and asthmatic Th2 reactions and a decrease in bactericidal properties of macrophages [110, 111]. The current concept says that the M1 phenotype can be reprogrammed to the M2 phenotype under the influence of RF-M2 and vice versa. However, it has been demonstrated on numerous occasions that the M1 phenotype can be transformed to the M2 phenotype under conditions of inflammation and the action of proinflammatory RF-M1, whereas the M2 phenotype can be transformed to the M1 phenotype under the influence of anti-inflammatory RF-M2.

Among the facts that contradict the current concept are the following:

1. The reprogramming of macrophages to the M2 phenotype in tumor-induced inflammation [17]. Such paradoxical reprogramming of macrophages promotes tumor survival and metastasis [112].
2. The reprogramming of macrophages from the M1 phenotype to M2 at the end of inflammation or in chronic inflammation [9], when the proinflammatory RF-M1s continue to affect macrophages.
3. The reprogramming of macrophages to the M2 phenotype* in vitro* under the influence of classic proinflammatory (according to the current concept) RF-M1 LPS [23].
4. Switching of macrophages from the M1 phenotype to the M2 phenotype* in vivo* under the influence of some LPS-containing microbes [9, 113]. Such reprogramming of macrophages helps bacteria to avoid the bactericidal action of the M1 macrophages.
5. The reprogramming of macrophages to the M1 phenotype and an increased production of proinflammatory cytokines under the influence of anti-inflammatory cytokines IL-10 and IL-13, which are under certain conditions, in fact RF-M2 [60, 114].
6. The reprogramming of macrophages to the M1 phenotype under the influence of a parasitic infection (RF-M2). Macrophages react to parasites by the formation of the M2 phenotype [8]. However, at the early stages of infection with* Taenia crassiceps*,* Schistosoma mansoni*, and* Trypanosoma congolense*, macrophages have the M1 phenotype and only at the later stages is the antiparasitic M2 phenotype formed [33, 115].
7. The existence of both M1 and M2 macrophages in the same microenvironment. Allergy and an allergy-associated increase in the production of the anti-inflammatory RF-M2 cytokines IL-4 and IL-13 program macrophages to the M2 phenotype [106]. However, in the area of the allergic inflammation, not only the M2 but also the M1 macrophages can be found [116]. In obesity, despite the prevalence of M1 macrophages among ATMs, M2 macrophages can be also found [117]. There is a similar situation in alcoholic hepatitis; namely, there are both M1 and M2 macrophages in the same microenvironment [118]. The current concept cannot explain how the macrophages belonging to alternative phenotypes could be found in the same microenvironment.

The current concept can explain how the M1 macrophages phenotype could be formed under the influence of RF-M1 and the M2 phenotype under the influence of RF-M2 [119]. However, the current concept can neither explain the facts listed above nor answer the question of how switching between different phenotypes under the influence of alternative RF occurs. In other words, how can the proinflammatory M1 phenotype be formed under the influence of the anti-inflammatory RF-M2 and, contrarily, how can the anti-inflammatory M2 phenotype be formed under the influence of the proinflammatory RF-M1?

### 3.2. The Interchange of the M1 and the M2 Phenotypes Might Well Occur as a Result of the Formation of the M3 Switching Phenotype

**Figure 10:**
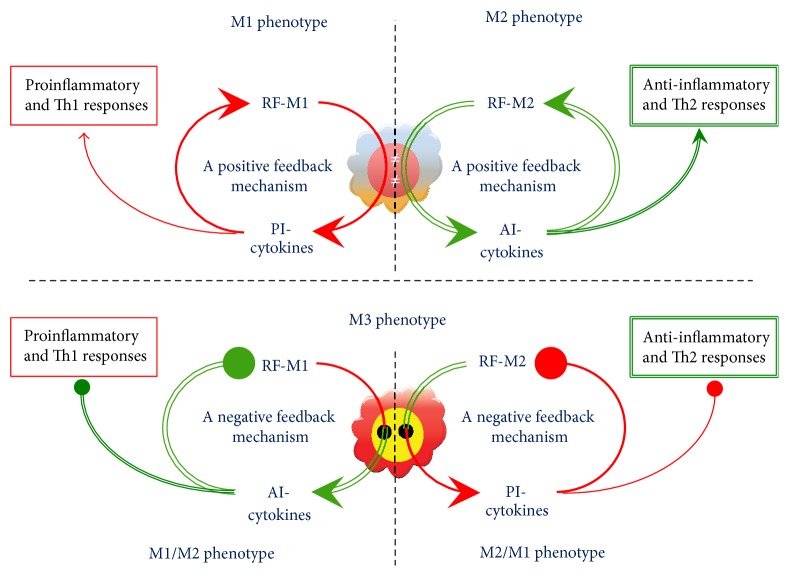
The hypothesis of the existence of the M3 phenotype: the switching phenotype. RF-M1: a reprogramming factor M1; RF-M2: a reprogramming factor M2, PI-cytokines: proinflammatory cytokines; AI-cytokines: anti-inflammatory cytokines. The M1 phenotype of macrophages upregulates the production of PI-cytokines in response to the RF-M1 resulting in a further shift towards M1; the M2 phenotype upregulates the production of AI-cytokines in response to the RF-M2 resulting in a further shift towards M2; the M3 phenotype (the switching phenotype) upregulates the production of AI-cytokines in response to the RF-M1 resulting in the reprogramming to the M2 phenotype (the M1/M2 phenotype) and, contrarily, the production of PI-cytokines in response to the RF-M2 resulting in the reprogramming to the M1 phenotype (the M2/M1 phenotype).

The aforementioned examples of paradoxical reprogramming of macrophages suggest a very interesting hypothesis: In addition to the M1 phenotype of macrophages, which in response to RF-M1 induces an increase in the production of the proinflammatory cytokines followed by a further shift towards the M1 phenotype, and the M2 phenotype, which in response to RF-M2 induces an increase in the production of the anti-inflammatory cytokines with a further shift to the M2 phenotype,* there is the third phenotype of macrophages*, which in response to RF-M1 induces an increase in the production of the anti-inflammatory cytokines and reprograms itself to the M2 phenotype and, contrarily, in response to RF-M2 induces an increase in the production of the proinflammatory cytokines and reprograms itself to the M1 phenotype (Figure 10). Such a phenotype could be referred to as the M3 phenotype or the switching phenotype. The M3 switching phenotype could be divided into two subtypes such as the M1/2 phenotype, which in response to RF-M1 induces the production of the M2 mediators, and the M2/1 phenotype, which in response to RF-M2 induces the production of the M1 mediators.

Experimental data indicate the existence of the M3 phenotype. Recently, we have obtained experimental data that support the hypothesis of the existence of the switching phenotype. We have found indicators of the formation of such a phenotype in lung diseases and during use of some medicines [120, 121].

We have found the phenotype of switching in lung diseases. Experiments have been carried out on alveolar macrophages isolated from patients suffering from chronic obstructive pulmonary disease (COPD) or bronchial asthma (BA) and on healthy control subjects. The macrophages were cultivated in RPMI-1640 medium with 10% fetal bovine serum (FBS). LPS was to activate the macrophages. A 0% FBS medium was used as RF-M1, whereas a 40% FBS medium was used as RF-M2 [14, 122]. The changes in phenotype were identified by the changes in the production of cytokines by the macrophages. In particular, an increased production of proinflammatory cytokines compared to 10% FBS indicated a shift of phenotype towards the M1 phenotype, whereas an increased production of anti-inflammatory cytokines indicated a shift towards the M2 phenotype.

We found that the macrophages from healthy subjects increased the production of the proinflammatory M1 cytokines and decreased the production of the anti-inflammatory M2 cytokines in response to RF-M1, whereas in response to RF-M2 the macrophages from healthy subjects increased the production of the anti-inflammatory M2 cytokines and decreased the production of the proinflammatory M1 cytokines. These changes in the production of specific cytokines reflect the normal adequate plasticity of macrophages from healthy subjects.

In contrast to the macrophages from healthy subjects, the macrophages from patients with COPD demonstrated a decrease in the production of the proinflammatory M1 cytokines INF-*γ*, IL-1*β*, and TNF-*α* in response to RF-M1, whereas in response to RF-M2 they demonstrated an increase in the production of the proinflammatory M1 cytokines TNF-*α* and IL-8. This situation could be termed as paradoxical/inverted reprogramming or paradoxical plasticity. Thus, alveolar macrophages from patients with COPD possessed the characteristics of the switching phenotype. There was no qualitative change in the reprogramming of macrophages in patients with BA in remission.

We have also found the phenotype of switching when prescribing inhaled glucocorticosteroids (IGCS). The effect of IGCS therapy on the macrophage phenotype and plasticity was evaluated in patients with BA. The results have shown that the macrophages from the IGCS-treated patients with BA decreased production of most proinflammatory M1 cytokines, such as IL-12, IFN-*γ*, IL-8, IL-6, and IL-1*β*, under normal conditions of cultivation with 10% FBS. However, macrophages from the IGCS-treated patients with BA increased production of the anti-inflammatory cytokine IL-10 compared to the macrophages from the healthy subjects and non-IGCS-treated patients with BA. These changes reflect the shift of the macrophage phenotype in patients with BA towards the anti-inflammatory M2 phenotype under the influence of IGCS. The same conclusion was made by others earlier [123].

Prescribing IGCS to patients with BA qualitatively affected their macrophage plasticity. In contrast to the healthy subjects and non-IGCS-treated patients with BA, the production of the proinflammatory cytokines IL-12, IFN-*γ*, IL-8, IL-6, and TNF-*α* by the macrophages from the IGCS-treated patients with BA decreased in response to RF-M1 and increased in response to RF-M2, whereas the production of the anti-inflammatory cytokines IL-4, IL-5, and IL-10 decreased in response to RF-M2 and increased in response to RF-M1. These changes in cytokine production suggest the formation of inverted plasticity of the macrophage secretory activity in the IGCS-treated patients with BA. Thus, the switching phenotype forms in response to IGCS.

### 3.3. The Issues Raised by the M3 Switching Phenotype

**Figure 11:**
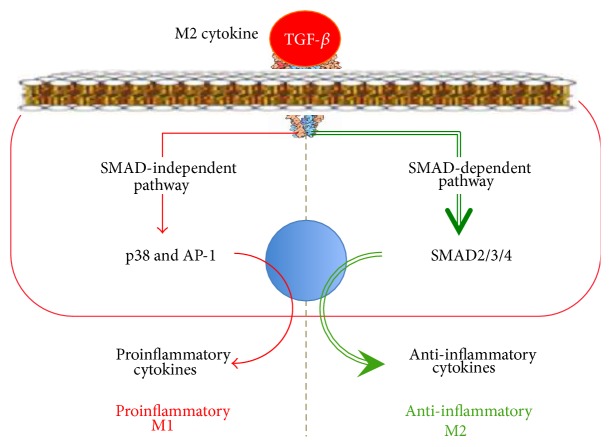
An offshoot of a signalling pathway resulting in the activation of an alternate M1 or M2 response is an important constructive feature of the intracellular pathways of macrophage reprogramming. The figure shows the offshoot of the TGF-*β*-dependent pathway of macrophage reprogramming. The signalling pathways transmitting the signal from TGF-*β* (RF-M2) and reprogramming macrophages to the anti-inflammatory M2 phenotype via SMAD2/3/4 possess the intracellular offshoot, which, in case of its activation, can upregulate the production of the proinflammatory M1 cytokines. Due to the offshoots of the intracellular signal pathways resulting in the production of alternate pro- and anti-inflammatory cytokines, macrophages can prevent an excessive inflammation and tissue damage during the reprogramming of macrophages to the M1 phenotype (e.g., when there is a need to eliminate a virus or bacterium) or a critical decrease in proinflammatory activity of macrophages and, therefore, their bactericidal function and the capacity to develop the autoimmune processes during the M2 phenotype reprogramming (e.g., when there is a need to kill extracellular parasites). SMAD2/3/4, p38, and AP-1: transcription factors.

An analysis of the literature and the results of our experiments suggest the existence of a new immunologic phenomenon, which could be referred to as inverted reprogramming or, in other words, paradoxical plasticity of macrophages. The data described above support the hypothesis of the existence of a particular macrophage phenotype, which increases the production of the M2 and decreases the production of the M1 cytokines in the proinflammatory microenvironment. On the contrary, this macrophage phenotype increases production of the M1 and decreases the production of the M2 cytokines in anti-inflammatory microenvironment. This hypothesis significantly extends the current concept of macrophage plasticity but immediately raises several important issues:

1. What role can the switching phenotype play in disease development and drug effects? Our data have shown that the switching phenotype is formed in lung diseases, for instance, in COPD [120]. Macrophages with the switching phenotype found in COPD did not increase but rather greatly decreased production of the proinflammatory cytokines in response to RF-M1. It could be suggested that such a phenotype promotes the decrease in bactericidal and antiviral activity of the lung immune system and the development of an increased susceptibility to lung infection. Therefore, this M3 phenotype may possibly play a pathogenic role in COPD. Furthermore, the M3 phenotype has been found in the alveolar macrophages from IGCS-treated patients with BA [121]. As IGCS improves the condition of patients with BA, the formation of the M3 phenotype might be suggested to be a component of the therapeutic action of IGCS. Despite the fact that this suggestion is still to be verified, it is already clear that the M3 phenotype could be considered as an effective therapeutic target worthy of attention.
2. Under what conditions can the M3 phenotype be formed? At present, there are no clear experimental or clinical data to answer this question. Nevertheless, based on the expediency of M3 phenotype formation, we suggest this phenotype would be formed when there is need for a negative feedback mechanism. When there is a danger of excessive inflammation as a result of the influence of RF-M1 on a macrophage, the M1/M2 is formed, whereas when there is a risk of a significant decrease in bactericidal or antiviral properties of macrophages as a result of the RF-M2 action, the M2/M1 is formed.
3. What intracellular mechanisms could be involved in the formation of the M3 phenotype? The findings presented above in The Reprogramming Signaling Pathways can help to answer this question, particularly the studies by Locati et al. [84] and by Zhou et al. [37]. Analysis of these studies has shown that there are seven intracellular signalling pathways involved in the reprogramming of macrophages, specifically the JNK-, PI3K/Akt-, Notch-, JAK/STAT-, TGF-*β*/SMAD/non-SMAD-, TLR/NF-*κ*B-, and hypoxia-induced signalling pathways. Almost every one of these pathways possesses an important constructive feature, namely, pathways, which transmit the signal from RF-M1 and, thereby, mediate the reprogramming of macrophages to the M1 phenotype; there is often an intracellular offshoot, which could result in an increase in the anti-inflammatory M2 cytokines. On the contrary, the M2 signalling pathways often have an offshoot which could result in an increase in the proinflammatory M1 cytokines (Figure 11). At the point of bifurcation, there is a protein switch that determines which way the reprogramming of macrophages will go. It is clear that such a protein (enzyme or transcription factor) is of the greatest importance for manipulating the macrophage phenotype in order to correct the immune response.

The existence of the offshoots of the reprogramming signalling pathways allows for understanding of how the M3 phenotype increases the production of the anti-inflammatory M2 cytokines and shifts the phenotype towards M2 in response to RF-M1 or, conversely, the M3 phenotype increases the production of the proinflammatory M1 cytokines and shifts the phenotype towards M1 in response to RF-M2.

### 3.4. A Possible Role of the M3 Phenotype in Immune Phenomena and Disease Development

The notion of M3 phenotype can be built perfectly into the concept of macrophage reprogramming and, thus, extends our understanding of the mechanisms of macrophage plasticity. A possible role of the M3 phenotype in immune phenomena and disease development can be demonstrated by the next examples.

*(1) Normal Termination of Inflammation and Immune Reactions Could Be Associated with the M3 Phenotype of Macrophages.* Excessive production of the proinflammatory or anti-inflammatory cytokines could lead to the development of proinflammatory diseases or to a great decrease in bactericidal, antiviral, and antitumoral activity of the immune system, respectively. The M3 phenotype could be suggested to help the immune system in resolving these issues. In inflammation, when the infection is already eliminated, but there are still many proinflammatory mediators in the microenvironment, in this case, the M1/M2 phenotype will promote termination of inflammation as a result of the negative feedback formation and increased production of the anti-inflammatory cytokines. Similarly, as a result of negative feedback, the M2/M1 phenotype can prevent a critical decrease in bactericidal, antiviral, and antitumoral functions of macrophages during the M2 phenotype reprogramming and development of an antiparasitic Th2 response. Indeed, the M2 phenotype can always be found in an area of decreased inflammation at the termination stage of inflammation [9], whereas, in an area of allergic reactions, not only the M2 but also the M1 phenotype can be found [116].

In fact, the results reported by Bystrom et al. [124] support similar conclusions. They have found that macrophages at normal termination of inflammation possess weak bactericidal activity compared to those at the initial stage of the inflammation. In other words, macrophages at the termination of inflammation manifest the features of the M1 and M2 phenotypes. The authors referred to this phenotype as the rM (resolution-phase macrophages) phenotype. Within the context of our hypothesis, the rM phenotype is one of the switching phenotypes, namely, the M1/M2 phenotype. 

*(2) The Pathogenesis of Many Diseases Is in Agreement with the Activity of the Switching Phenotype.* For instance, the switching phenotype could be involved in the reprogramming of the pathologic M2 phenotype under conditions when many proinflammatory mediators are in the tumor microenvironment. In particular, proinflammatory TNF-*α* has been found to demonstrate the greatest concentration increase in mice peritoneal ascites with Ehrlich carcinoma [125], and this increase is accompanied by macrophages having the M2 phenotype (our unpublished data). It could be suggested that the formation of TAM-M2 in tumoral inflammation is associated with formation of the switching phenotype M1/M2.

In addition, the pathogenesis of inflammatory diseases could be suggested to be associated with a disturbance in the formation of the M3 switching phenotype. In particular, in patients with a chronic venous ulcer, a disturbed process of switching from the macrophage M1 phenotype to the M2 was observed [126]. Also, our data suggest that the M3 phenotype is involved in the development of COPD. 

*(3) The Switching Phenotype May Be Involved in the Formation of the Phenomenon of LPS Tolerance.* The existence of several isoforms of Akt allows macrophages to be reprogrammed to both the M1 and M2 phenotype via the PI3K/Akt-signalling pathway in response to the same ligand of LPS [22, 23, 41, 127]. This makes it clear that the “LPS-tolerant” phenotype is not a strict definition of the phenotype that decreases production of the proinflammatory cytokines and increases production of anti-inflammatory cytokines in response to the M1-inductor LPS. This phenotype does not become less sensitive to LPS; that is, it does not become LPS tolerant. This phenotype just changes its reaction to LPS. It has been established that the p50/р50 NF-*κ*B homodimers, which prevent activation of the M1 phenotype genes and activate the M2 phenotype genes, are accumulated in the nuclei of the “LPS-tolerant” macrophages [76]. Within the context of our hypothesis, the “LPS-tolerant” phenotype is the switching phenotype M1/M2. 

*(4) The Switching Phenotype Could Be Involved in the Defence Strategy of Some Bacteria and Viruses.* Most viruses and bacteria reprogram macrophages to the M1 phenotype [10], and then the M1 macrophages eliminate the infection. However, some microbes can suppress the bactericidal and antiviral properties of the innate immunity due to reprogramming of macrophages to the M2 phenotype [9, 113, 127]. This mechanism of microbial defence strategy could be suggested to involve the formation of the macrophage switching phenotype M1/M2, which, instead of producing bactericidal free radicals and proinflammatory cytokines, produces anti-inflammatory cytokines in response to microbes.

Among immune cells, there are some cells which behave similarly to the macrophages with the switching phenotype. These are Treg lymphocytes, which are known to produce anti-inflammatory cytokines in response to a proinflammatory microenvironment [128, 129]. In fact, the Treg lymphocytes have a switching phenotype as proposed for macrophages. The existence of Treg lymphocytes proves the possibility that intracellular mechanisms could form a switching phenotype in cells.

## 4. Conclusions

The current concept of macrophage reprogramming describes the dynamics of changes from the M1 to M2 phenotype as a “continuum” [1, 17, 80], that is, as a gradual and continuous process. The concept of a continuum is obviously valid in a situation when the phenotype changes from M1 to M2 in response to RF-M2 or when it changes from M2 to M1 in response to RF-M1. The situation when the phenotype changes from M1 to M2 or, contrarily, from M2 to M1 in response to the alternative reprogramming microenvironment (RF-M1 or RF-M2, resp.) obviously does not comply with the concept of a continuum. Our hypothesis suggests that the changes in phenotype from M1 to M2 and vice versa could occur via the formation of the M3 switching phenotype. If this is so, then the changes of phenotype should be described using the term of “discretuum.”

Our hypothesis implies a new understanding of macrophage plasticity and allows us to answer the question of how the paradoxical reprogramming of these cells occurs, that is, how the anti-inflammatory M2 phenotype is formed in response to RF-M1 in inflammation and, contrarily, how the proinflammatory M1 phenotype is formed in response to RF-M2. The understanding of the mechanism of the paradoxical reprogramming of macrophages will extend our vision of the mechanisms of plasticity of the immune response. This understanding will also help to answer several important questions of modern immunology and medicine:

1. How does the TAM reprogramming to the M2 phenotype occur in tumor-induced inflammation [17, 119]?
2. How does the reprogramming of macrophages from the M1 to M2 phenotype occur in normal termination of an inflammation [8] at the stage when macrophages are still affected by the proinflammatory RF-M1?
3. How does the formation of the M2 phenotype in response to an RF-M1, such as LPS [23], occur?
4. How do some bacteria switch the macrophage phenotype from M1 to M2 [9, 113], so as to avoid the bactericidal action of the M1 macrophages?
5. How can the anti-inflammatory M2 cytokines IL-10 and IL-13 program the M1 phenotype and increase the production of the proinflammatory M1 cytokines [60, 114]?
6. Why can the alternate macrophage phenotypes be found in the same microenvironment during the development of allergic reactions and in obesity and alcoholic hepatitis [116–118]?
7. How is the phenomenon of LPS tolerance formed?
8. Why do some inflammatory diseases develop?
9. What is the new mechanism of the action of glucocorticoids?

It seems evident that the switching phenotype may potentially play a significant role during the development of inflammatory, autoimmune, oncologic, and other diseases. Thus, the switching phenotype should be a therapeutic target in the development of new medicines and medical cell technologies for treating diseases associated with a disturbed immune response.
